# Identification of *miR-199-5p* and *miR-199-3p* Target Genes: Paxillin Facilities Cancer Cell Aggressiveness in Head and Neck Squamous Cell Carcinoma

**DOI:** 10.3390/genes12121910

**Published:** 2021-11-27

**Authors:** Nozomi Tanaka, Chikashi Minemura, Shunichi Asai, Naoko Kikkawa, Takashi Kinoshita, Sachi Oshima, Ayaka Koma, Atsushi Kasamatsu, Toyoyuki Hanazawa, Katsuhiro Uzawa, Naohiko Seki

**Affiliations:** 1 Department of Oral Science, Graduate School of Medicine, Chiba University, Chiba 260-8670, Japan; n.tanaka@chiba-u.jp (N.T.); minemura@chiba-u.jp (C.M.); Sachi.o8952@chiba-u.jp (S.O.); axna4812@chiba-u.jp (A.K.); kasamatsua@faculty.chiba-u.jp (A.K.); uzawak@faculty.chiba-u.jp (K.U.); 2 Department of Functional Genomics, Graduate School of Medicine, Chiba University, Chiba 260-8670, Japan; cada5015@chiba-u.jp (S.A.); naoko-k@hospital.chiba-u.jp (N.K.); 3 Department of Otorhinolaryngology, Head and Neck Surgery, Graduate School of Medicine, Chiba University, Chiba 260-8670, Japan; t.kinoshita@chiba-u.jp (T.K.); thanazawa@faculty.chiba-u.jp (T.H.)

**Keywords:** microRNA, HNSCC, *miR-199-5p*, *miRNA-199-3p*, paxillin, TCGA

## Abstract

Our previous study revealed that the *miR-199* family (*miR-199a-5p*/*-3p* and *miR-199b-5p*/*-3p*) acts as tumor-suppressive miRNAs in head and neck squamous cell carcinoma (HNSCC). Furthermore, recent studies have indicated that the passenger strands of miRNAs are involved in cancer pathogenesis. The aim of this study was to identify cancer-promoting genes commonly regulated by *miR-199-5p* and *miR-199-3p* in HNSCC cells. Our in silico analysis and luciferase reporter assay identified paxillin (*PXN*) as a direct target of both *miR-199-5p* and *miR-199-3p* in HNSCC cells. Analysis of the cancer genome atlas (TCGA) database showed that expression of *PXN* significantly predicted a worse prognosis (5-year overall survival rate; *p* = 0.0283). *PXN* expression was identified as an independent factor predicting patient survival according to multivariate Cox regression analyses (*p* = 0.0452). Overexpression of PXN was detected in HNSCC clinical specimens by immunostaining. Functional assays in HNSCC cells showed that knockdown of *PXN* expression attenuated cancer cell migration and invasion, suggesting that aberrant expression of *PXN* contributed to HNSCC cell aggressiveness. Our miRNA-based approach will provide new insights into the molecular pathogenesis of HNSCC.

## 1. Introduction

Global cancer statistics in 2018 stated that head and neck squamous cell carcinoma (HNSCC) was the eighth most common human malignancy worldwide [1]. Every year, there are approximately more than 800,000 new HNSCC cases diagnosed and 430,000 deaths from HNSCC [2]. HNSCC arises from the oral cavity, hypopharynx, nasopharynx, and larynx, and the most common subtype of HNSCC is oral squamous cell carcinoma (OSCC) [3]. There are regional differences in the frequency of HNSCC occurrence [4], and India and Sri Lanka are high-risk countries for HNSCC, accounting for more than 20% of all cancers [5].

Approximately 60% of patients with HNSCC are at an advanced stage at the time of diagnosis [6]. Aggressive progression of HNSCC is characterized by high rates of recurrence, distant metastasis, and drug resistance acquired by cancer cells during treatment [7]. To date, a number of treatment strategies, such as radiotherapy, chemoradiotherapy, molecular targeted agents and immune checkpoint inhibitors have been devised and implemented for HNSCC [8]. However, the treatment outcomes of HNSCC have not improved significantly in recent decades, and the molecular mechanisms of malignant transformation of HNSCC are not completely understood.

A vast number of studies indicate that non-coding RNAs are involved in several biological processes, e.g., cell proliferation, apoptosis, development, epithelial-to-mesenchymal transition (EMT), and chromatin remodeling [9,10]. In human cancers, aberrant expression of non-coding RNAs closely contributes to malignant transformation, metastasis, and drug resistance [11]. However, the biological role and functions of non-coding RNAs are still mostly uncharacterized.

MicroRNA (miRNA) is small non-coding RNA that regulates the expression of RNA transcripts in normal and disease cells in a sequence-dependent manner [12]. A unique property of miRNAs is that a single miRNA can control a vast number of genes in a cell. In addition, the genes regulated by miRNAs vary from cell to cell. Therefore, aberrant expression of miRNAs disrupts intracellular transcriptional networks, which, in turn, causes human diseases, including cancers [13]. A growing body of evidence suggests that large non-coding RNAs act as miRNA sponges, reducing their regulatory effect on mRNAs [14]. It is likely that complex transcriptional regulatory networks involving long non-coding RNAs, miRNAs and protein-coding RNAs exist in cells. Elucidation of these complex RNA networks will lead to an understanding of the molecular pathogenesis of human cancers.

In the human genome, there are multiple miRNAs with the same mature miRNA sequence located at different positions on chromosomes; these constitute an miRNA family. Analysis of our miRNA signatures, determined by RNA sequencing, showed that the *miR-199* family is frequently downregulated in a wide range of cancer tissues [15]. The *miR-199* family is composed of three members, *miR-199a-1*, *miR-199a-2*, and *miR-199b*, located on human chromosomes 19p13.2, 1q24.3, and 9q34.11, respectively. In addition, two mature miRNAs (the guide strand and passenger strand) are derived from each pre-miRNA; thus, there are six miRNAs in the *miR-199* family. The guide strands of the *miR-199* family share the same seed sequence (CCAGUGU), and the passenger strands share the seed sequence (CAGUAGU). Analysis of our miRNA expression signatures of HNSCC and OSCC, determined by RNA sequencing, showed that the *miR-199* family is downregulated in cancer tissues [15,16,17]. Our previous study revealed that the *miR-199* family members are downregulated in HNSCC tissues, and ectopic expression of these miRNAs markedly attenuated cancer cell migration and invasive abilities [16]. It is important to identify the genes regulated by miRNAs in different cancer types. The aim of this study was to identify the *miR-199* gene targets closely involved in HNSCC oncogenesis. Our *in silico* analysis revealed that a total of 12 genes (*ABCA1*, *ADRBK2*, *ANKRD52*, *DEPDC1B*, *FXR1*, *ITGA3*, *KLF12*, *NLK*, *PCDH17*, *PDE7A*, *PXN*, and *SLC24A2*) are regulated by *miR-199-5p* and *miR-199-3p* in HNSCC cells. Among these targets, high expression of paxillin (*PXN*) and FMR1 autosomal homolog 1 (*FXR1*) significantly predicted the 5-year overall survival rate of patients with HNSCC. Furthermore, we demonstrated that aberrant expression of *PXN* facilitated cancer cell migration and invasion.

## 2. Materials and Methods

### 2.1. Identification of Putative Targets Controlled by miR-199-5p and miR-199-3p in HNSCC Cells

**Figure 1:**
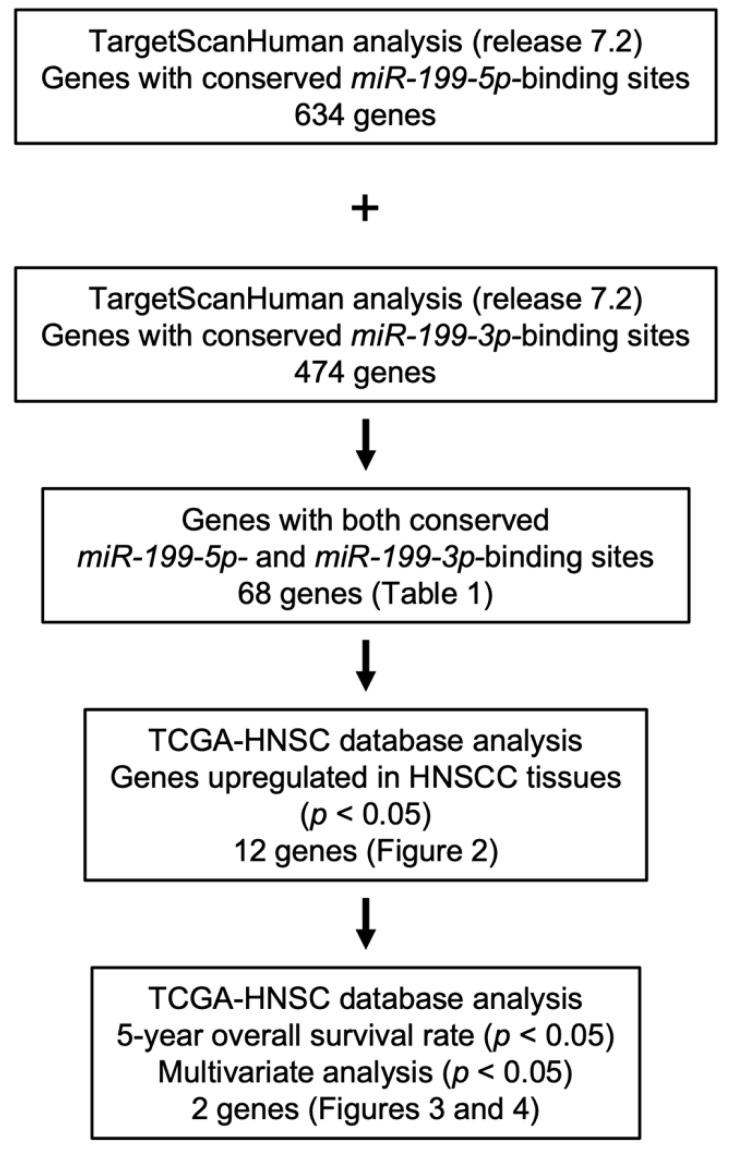
Flowchart of the strategy used to identify candidate *miR-199-5p* and *miR-199-3p* target genes in HNSCC cells.

The sequences of members of the *miR-199* family were confirmed using miRbase ver. 22.1 (https://www.mirbase.org, accessed on 10 April 2020) [18]. A flowchart of the procedure used to identify common target genes of *miR-199-5p* and *miR-199-3p* in this study is shown in Figure 1. We selected putative target genes that had both *miR-199-5p* and *miR-199-3p*-binding sites using TargetScanHuman ver. 7.2 (http://www.targetscan.org/vert_72/; data downloaded on 10 July 2020) [19]. The candidate genes were narrowed down by analyzing HNSCC clinical information obtained from The Cancer Genome Atlas (TCGA-HNSC).

For the Kaplan–Meier survival analysis and multivariate Cox regression analysis, we downloaded TCGA-HNSC clinical data (TCGA, Firehose Legacy) from cBioportal (https://www.cbioportal.org, accessed on 10 April 2020). Expression data for each gene were collected from OncoLnc (http://www.oncolnc.org; accessed on 20 April 2021) [20]. Patients from TCGA-HNSC were divided into low and high *FXR1*/*PXN* expression groups based on the median gene expression level, and the log-rank test was performed to compare the 5-year overall survival rate between the groups. In addition, Cox proportional hazards regression was conducted, using gene expression levels, tumor stage, pathological grade, and age as covariates. 

We used JMP Pro 15 (SAS Institute Inc., Cary, NC, USA) to perform the log-rank test and Cox proportional hazards regression.

### 2.2. Gene set Enrichment Analysis (GSEA)

To analyze the molecular pathways related to *FXR1* and *PXN* (regulated by *miR-199a-5p* and *3p*), we performed GSEA. Using TCGA-HNSC data, we divided HNSCC patients into high and low expression groups according to the Z-score of the *FXR1* or *PXN* expression level. A ranked list of genes was generated by log2 ratio comparing the expression levels of each gene between the two groups. The genes upregulated in the high expression groups were ranked higher. The obtained gene lists were uploaded into GSEA software [21,22]. We used KEGG subset of canonical pathways in The Molecular Signatures Database [21,23].

### 2.3. HNSCC Cell Lines

The HNSCC cell lines (SAS and Sa3) used in this study were obtained from the RIKEN BioResource Center (Tsukuba, Ibaraki, Japan). The characteristics of the cell lines are shown in Supplementary Table S1.

### 2.4. Transfection of Mature miRNAs and Small-Interfering RNAs (siRNAs) into HNSCC Cells

The miRNA precursors, negative control miRNA, and siRNAs were obtained from Invitrogen/Thermo Fisher Scientific (Waltham, MA, USA). The procedures used for transient transfection of miRNAs, siRNAs, and plasmid vectors have been described in our previous studies [16,17,24]. The reagents used are listed in Supplementary Table S2. All miRNAs and siRNA were transfected into HNSCC cell lines at 10 nM using RNAiMAX.

### 2.5. RNA Extraction and Quantitative Reverse-Transcription PCR (qRT-PCR)

RNA extraction from HNSCC cell lines and qRT-PCR were performed as described in our previous studies [16,17,24]. Briefly, total RNA was harvested using Trizol reagent and the PureLink™ RNA Mini Kit (Invitrogen/Thermo Fisher Scientific, Waltham, MA, USA). Reverse transcription was performed using the High-Capacity cDNA Reverse Transcription Kit (Applied Biosystems, Waltham, MA, USA). qRT-PCR was performed using the StepOnePlus™ Real-Time PCR System (Applied Biosystems). *GAPDH* was used as the internal control.

### 2.6. Immunohistochemistry

The immunohistochemistry procedures have been described in our previous studies [16,17,24]. Briefly, paraffin sections of tumors were obtained from HNSCC patients who underwent surgical treatment at Chiba University hospital. The clinical features of the HNSCC patients are summarized in Table S3. Our study was approved by the Ethics Committee of Chiba University (approval number: 28–65, 10 February 2015). Specimens were incubated with primary anti-PXN antibody overnight at 4 °C, after which they were incubated with DAKO Env+ secondary antibody for 30 min, washed, and counterstained with hematoxylin.

### 2.7. Western Blotting

The Western blotting procedures have been described in our previous studies [16,17,24]. Briefly, cell lysates were loaded onto 4–15% polyacrylamide gels at 18 μg/well. The proteins were transferred to polyvinylidene fluoride membranes and incubated with the primary antibody overnight at 4 °C and with the secondary antibody for 1 h. GAPDH was used as internal control.

### 2.8. Dual Luciferase Reporter Assays

A *PXN* DNA sequence with or without the miRNA-binding sequence of interest in its 3′ untranslated region (UTR) was inserted into the psiCHECK-2 vector (C8021; Promega, Madison, WI, USA). The plasmid vectors were then transfected into cells using Lipofectamine 2000 (Invitrogen, Waltham, MA, USA) at a final concentration of 50 ng/well. After 48 h of transfection, dual luciferase reporter assays were performed using the Dual Luciferase Reporter Assay System (Promega, Madison, WI, USA). Normalized data are expressed as the Renilla/Firefly luciferase activity ratio.

### 2.9. Cell Proliferation, Migration, and Invasion Assays in HNSCC Cells

The XTT assay to assess cell proliferation, and the Matrigel chamber assay to assess invasion, were performed in HNSCC cells as described previously [16,17,24]. In the wound healing assay, to assess migration a wound was created using a micropipette tip in the middle of each plate of cells transfected with siRNAs for 48 h. We incubated the plates at 37 °C and captured live cell migration after 6 and 12 h.

### 2.10. Statistical Analysis

Statistical analyses were performed using JMP Pro 15 (SAS Institute Inc., Cary, NC, USA). Differences between two groups were evaluated by Welch’s *t*-test. Dunnett’s test was used for multiple group comparisons. A *p*-value < 0.05 was considered statistically significant. The bar graphs present means and standard errors. 

## 3. Results

### 3.1. Identification of miR-199-5p and miR-199-3p Coordinately Regulated Genes in HNSCC Cells

Chromosomal locations and mature sequences of the miR-199 family are shown in Supplementary Figure S1. In humans, the miR-199 family is composed of three members, miR-199a-1, miR-199a-2, and miR-199b, located on human chromosomes 19p13.2, 1q24.3, and 9q34.11, respectively. The guide strands of the miR-199 family share the same seed sequence (CCAGUGU); the passenger strands share same sequence (CAGUAGU).

TCGA database analysis showed the downregulation of all members of the miR-199 family in HNSCC clinical tissues (Supplementary Figure S2). At first, we confirmed tumor-suppressive functions of miR-199-5p and miR-199-3p in HNSCC cells by ectopic expression assays. Expression of miR-199-5p and miR-199-3p did not affect cell proliferation in SAS and Sa3 cells (Supplementary Figure S3A). Cell invasion and migration features were markedly suppressed by miR-199-5p and miR-199-3p expression in SAS and Sa3 cells (Supplementary Figure S3B,C). Typical images of cells from the invasion and migration assays are shown in Supplementary Figures S4 and S5. These data clearly show that both miR-199-5p and miR-199-3p acted as tumor-suppressive miRNAs in HNSCC cells.

The flowchart in Figure 1 shows the procedure we used to identify target genes of *miR-199-5p* and *miR-199-3p*. First, we searched for putative *miR-199-5p-* and *miR-199-3p*-binding genes in the TargetScan database (release 7.2). We found 634 and 474 transcripts that contained conserved *miR-199-5p-* and *miR-199-3p*-binding sites in their 3′UTRs, respectively. Of these transcripts, 68 transcripts possessed putative binding sites for both *miR-199-5p* and *miR-199-3p* (Table 1).

### 3.2. Expression and Clinical Significance of the Putative Target Genes in Patients with HNSCC according to TCGA Analysis

**Figure 2:**
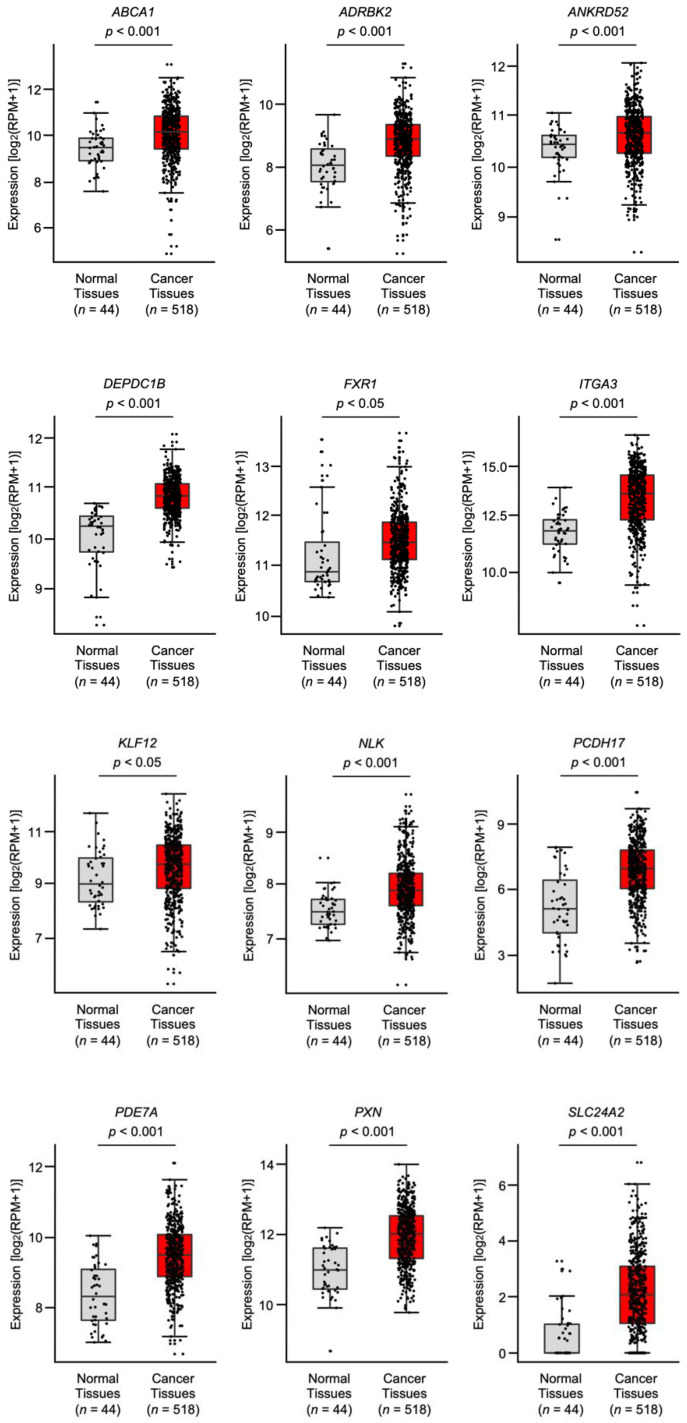
HNSCC tissue expression of 12 target genes with *miR-199-5p-* and *miR-199-3p*-binding sites in their 3’UTRs using TCGA-HNSC data. The expression levels of 12 genes (*ABCA1*, *ADRBK2*, *ANKRD52*, *DEPDC1B*, *FXR1*, *ITGA3*, *KLF12*, *NLK*, *PCDH17*, *PDE7A*, *PXN*, and *SLC24A2*) were analyzed using TCGA-HNSC database. A total of 518 HNSCC tissues and 44 normal epithelial tissues were evaluated.

The expression levels of the putative targets (68 genes) of *miR-199-5p* and *miR-199-3p* were evaluated using the TCGA database (TCGA-HNSC). Among these genes, the expression levels of 12 genes (*ABCA1, ADRBK2, ANKRD52, DEPDC1B, FXR1, ITGA3, KLF12, NLK, PCDH17, PDE7A, PXN*, and *SLC24A2*) were significantly upregulated in HNSCC tissues (*n* = 518) compared with normal tissues (*n* = 44) (Figure 2). Among these 12 genes, *DEPDC1B* had a negative correlation with the *miR-199* family in cancer tissues according to Spearman’s rank test (Supplementary Figure S6).

**Figure 3:**
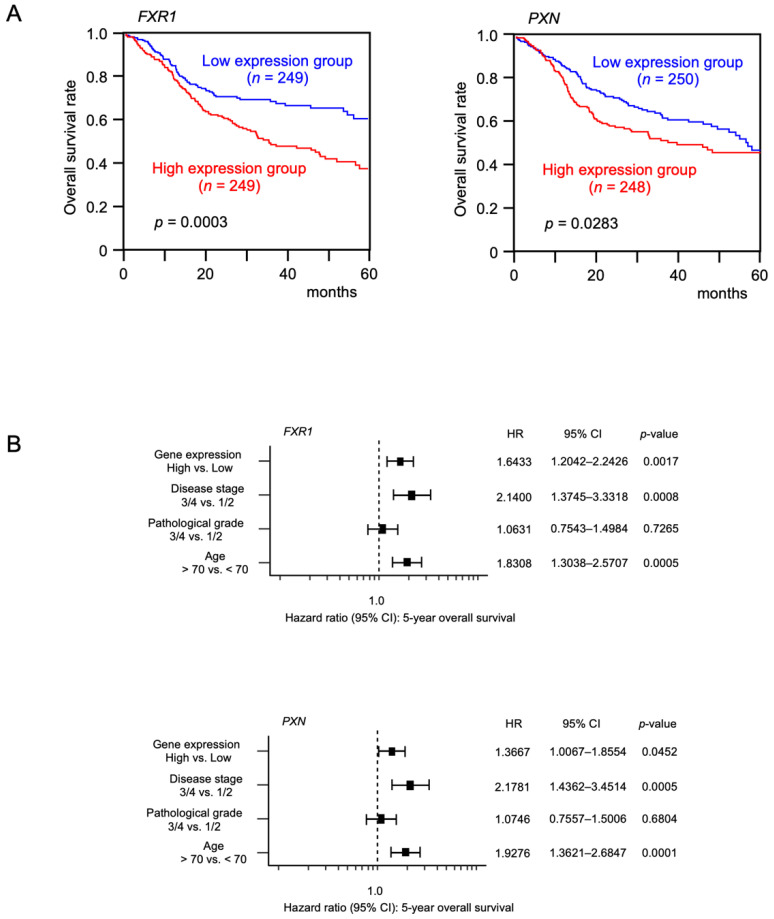
Clinical significance of FXR1 and PXN using TCGA-HNSC data. (**A**) Kaplan–Meier survival analyses of HNSC patients using data from TCGA database. Patients were divided into high and low expression groups according to the median FXR1 and PXN expression levels. The red and blue lines indicate the high and low expression groups, respectively. (**B**) Forest plot showing the multivariate analysis results for two genes (FXR1 and PXN), which were identified as independent prognostic factors for overall survival after adjustment for patient age, disease stage, and pathological grade.

To determine clinical relevance, the 5-year overall survival rates of HNSCC patients according to the expression levels of these 12 genes were determined using TCGA-HNSC data. Patients with high expression of either *FXR1* (*p* = 0.0003) or *PXN* (*p* = 0.0283) had a significantly worse prognosis compared with those with low expression (Figure 3). Moreover, multivariate Cox regression analysis revealed that the expression levels of *FXR1* (*p* = 0.0017) and *PXN* (*p* = 0.0452) were independent prognostic factors in patients with HNSCC (Figure 3).

### 3.3. FXR1- and PXN-Mediated Pathways in HNSCC Cells

We investigated the genes differentially expressed between the high and low *FXR1* or *PXN* expression groups in the HNSCC cohort from TCGA using GSEA. A total of four and fourteen gene sets were significantly enriched (FDR *q*-value < 0.05) in the high *FXR1* and *PXN* expression groups, respectively (Table 2B). The most enriched gene set in the high *FXR1* expression group was the cardiac muscle contraction KEGG pathway, whereas that in the high *PXN* expression group was the focal adhesion KEGG pathway. Although both *FXR1* and *PXN* were involved in the prognosis of HNSCC, based on our in silico analyses, the results of GSEA analysis suggested that FXR1 was mainly involved in the pathogenesis of myocardial function. HNSCC is characterized by high rates of local recurrence and distant metastasis. Therefore, we focused on the roles of *PXN* in the KEGG pathways of focal adhesion and ECM receptor interaction, which are closely associated with local recurrence and distant metastasis, by performing functional analyses.

### 3.4. Expression of PXN in HNSCC Clinical Specimens

**Figure 4:**
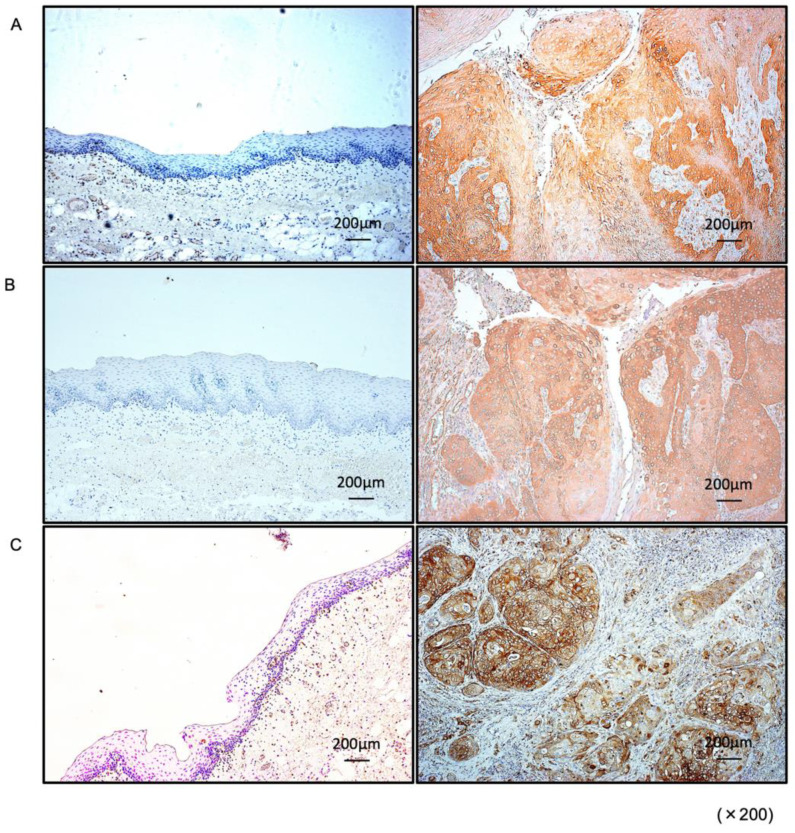
Overexpression of PXN in HNSCC clinical specimens. (**A**–**C**) Immunohistochemical staining of PXN in HNSCC clinical specimens. PXN expression was high in the nuclei and/or cytoplasm of cancer cells (right panels) but weak in normal mucosa (left panels).

Expression of PXN protein was investigated by immunostaining in HNSCC clinical specimens. Aberrant expression of PXN was detected in HNSCC lesions (Figure 4). In contrast, there was almost no PXN expression in the normal epithelium (Figure 4).

### 3.5. Regulation of PXN Expression by miR-199-5p and miR-199-3p in HNSCC Cells

**Figure 5:**
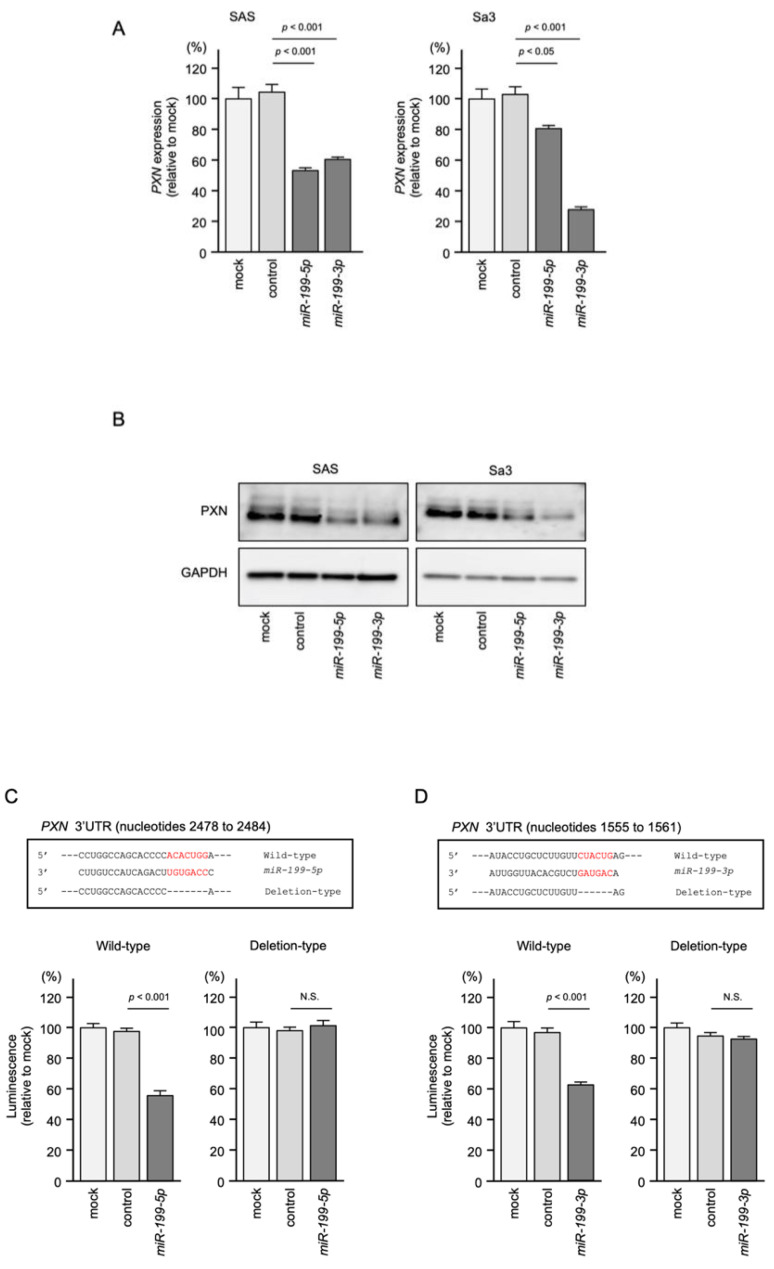
Direct regulation of *PXN* expression by both *miR-199-5p* and *miR-199-3p* in HNSCC cells. (**A**) qRT-PCR showing significantly reduced expression of *PXN* mRNA at 72 h after *miR-199-5p* or *miR-199-3p* transfection in SAS and Sa3 cells. Expression of *GAPDH* was used as an internal control. (**B**) Western blot showing reduced expression of PXN protein at 72 h after *miR-199-5p* or *miR-199-3p* transfection in SAS and Sa3 cells. Expression of GAPDH was used as an internal control. (**C**) TargetScan database analysis predicting a single putative *miR-199-5p*-binding site in the 3′-UTR of *PXN* (upper panel). Dual luciferase reporter assays showed reduced luminescence activity after cotransfection of the wild-type vector and *miR-199-5p* in SAS cells (lower panel). Normalized data are expressed as the Renilla/Firefly luciferase activity ratio (N.S., not significant). (**D**) TargetScan database analysis predicting a single putative *miR-199-3p*-binding site in the 3′-UTR of *PXN* (upper panel). Dual luciferase reporter assays showed reduced luminescence activity after cotransfection of the wild-type vector and *miR-199-3p* in SAS cells (lower panel).

Both the mRNA and protein levels of PXN were reduced after *miR-199-5p* and *miR-199-3p* transfection in SAS and Sa3 cells (Figure 5A,B). Full-size Western blots are shown in Supplementary Figure S7.

To investigate whether *miR-199-5p* and *miR-199-3p* bind directly to *PXN* in HNSCC cells, we conducted a dual-luciferase reporter assay. Luciferase activity was significantly reduced following cotransfection with *miR-199-5p* and a vector containing the *miR-199-5p*-binding site in the 3′UTR of *PXN*. On the other hand, cotransfection with a vector containing the *PXN* 3′UTR in which the *miR-199-5p*-binding site was deleted resulted in no change in luciferase activity (Figure 5C).

Similar to *miR-199-5p*, luciferase activity was significantly reduced following cotransfection with *miR-199-3p* and a vector containing the *miR-199-3p*-binding site in the 3′UTR of *PXN*, but not with a vector lacking the *miR-199-3p*-binding site in the *PXN* 3′UTR (Figure 5D).

These findings suggest that *miR-199-5p* and *miR-199-3p* bind directly to the 3′UTR of *PXN* and regulate the expression of *PXN* in HNSCC cells.

### 3.6. Effects of PXN Knockdown on the Proliferation, Invasion and Migration of HNSCC Cells

To assess the tumor-promoting effect of *PXN* in HNSCC cells, we performed knockdown assays using siRNAs. First, the inhibitory effects of three different siRNAs targeting PXN (si*PXN*-1, si*PXN*-2 and si*PXN*-3) on *PXN* expression were examined. The *PXN* mRNA and protein levels were effectively suppressed after transfection of each siRNA into SAS and Sa3 cells (Supplementary Figure S8A,B). For the subsequent functional assays in HNSCC cells, we used si*PXN*-1 and si*PXN*-3.

**Figure 6:**
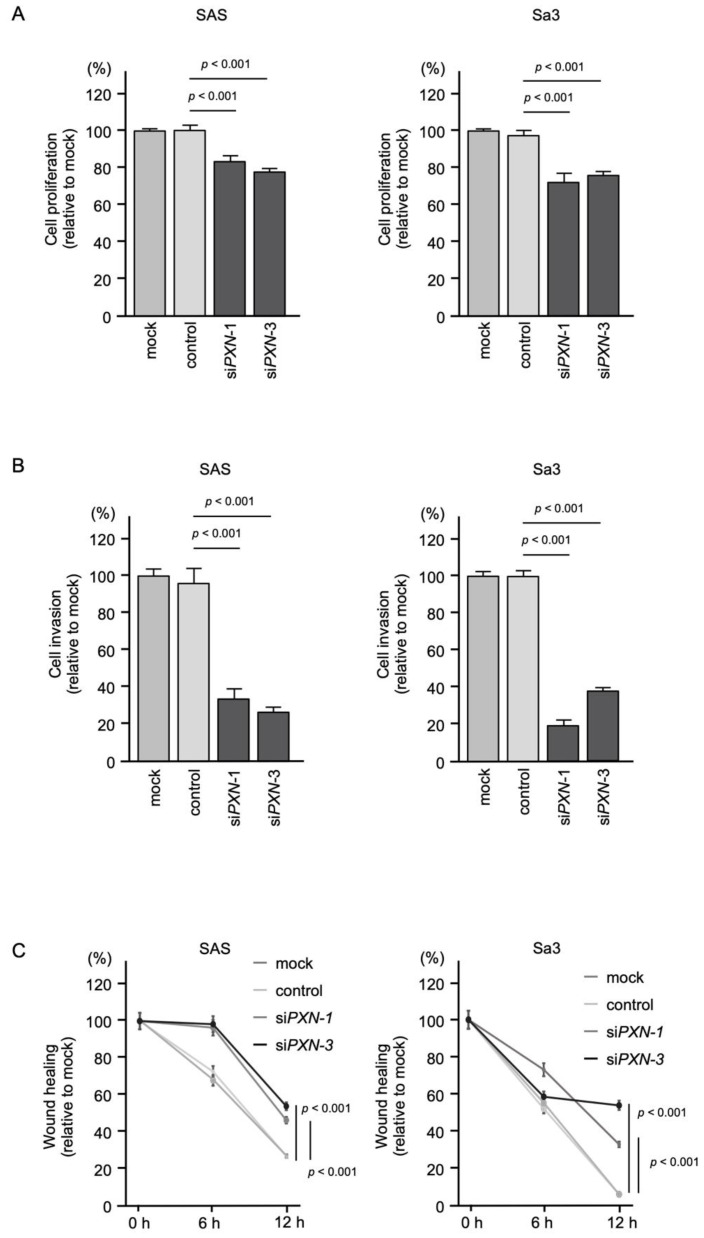
Functional assays of cell proliferation, invasion and migration following transient transfection of siRNAs targeting *PXN* in HNSCC cell lines (SAS and Sa3 cells). (**A**) Cell proliferation assessed by XTT assay at 72 h after siRNA transfection. (**B**) Cell invasion assessed by Matrigel invasion assays at 48 h after seeding siRNA-transfected cells into chambers. (**C**) Cell migration assessed by wound healing assay at 0, 6, and 12 h after cell scratch formation.

Knockdown of *PXN* had a slight inhibitory effect on cell proliferation in SAS and Sa3 cells (Figure 6A), and cell invasion and migration were significantly inhibited after si*PXN*-1 and si*PXN*-3 transfection in SAS and Sa3 cells (Figure 6B,C). Experimental photographs of typical results from the invasion and wound healing assays are shown in Supplementary Figures S9 and S10.

These findings indicate that overexpression of *PXN* is involved in promoting cancer cell migration and invasion.

## 4. Discussion

Currently available RNA sequencing technologies are suitable for creating miRNA expression signatures in various types of human cancers [25]. Analysis of our miRNA expression signatures in cancers revealed that some passenger strands of miRNAs derived from pre-miRNAs (e.g., *miR-31-3p*, *miR-145-3p*, *miR-99a-3p*, and *miR-139-3p*) are dysregulated in HNSCC tissues. Moreover, our functional assays showed that these passenger strands contribute to HNSCC oncogenesis, and that several of their target genes are closely associated with HNSCC pathogenesis [17,24,26,27].

Recently, a large number of TCGA cohort studies showed that certain miRNAs (e.g., both the 5p and 3p strands of *miR-29*, *miR-30a*, *miR-143*, *miR-145*, and *miR-139*) coordinately regulate oncogenic pathways in cancer cells [28]. In opposition to the traditional concept of miRNA biogenesis, the involvement of miRNA passenger strands in cancer pathogenesis is a newly proposed concept. Therefore, searching for target genes of miRNA passenger strands, as well as guide strands, is an important issue in cancer research.

Previous studies have reported that *miR-199* family members are closely implicated in a variety of cancers as either tumor-suppressors or oncogenes [29]. Expression of *miR-199a-5p* was significantly reduced in OSCC tissues, and its overexpression blocked the EMT cascade by targeting *SOX4* [30]. Another study showed that transient transfection of *miR-199a-5p* suppressed the malignant phenotypes of OSCC cells, for example, blocking cell proliferation and inducing G0/G1 arrest and apoptosis. This study indicated that IKKb, a pivotal activator of NF-κB, is a direct target of *miR-199a-5p* [31].

Our next focus was to identify oncogenic targets regulated by both *miR-199-5p* and *miR-199-3p* in HNSCC cells. Our in silico analysis revealed that *FXR1* and *PXN* are candidate targets of *miR-199-5p* and *miR-199-3p* in HNSCC cells. Importantly, expression of *FXR1* and *PXN* was closely associated with the molecular pathogenesis of HNSCC.

Overexpression of FXR1, an RNA-binding protein, has been reported in a wide range of cancers, including HNSCC and oral cancer [32,33,34,35]. Previous studies showed that overexpressed FXR1 promoted cancer cell aggressiveness by binding to and disrupting p21 mRNA [36]. Interestingly, in oral cancer cells, FXR1 stabilized *miR-301a-3p*, and these events reduced expression of p21 [37]. How aberrantly expressed FXR1 is involved in miRNA networks in HNSCC cells is an important question.

*PXN* is an intercellular adaptor protein that interacts with multiple proteins involved in cell adhesion [38]. Notably, PXN can connect integrins to focal adhesion kinase (FAK) and is a pivotal player in the assembly and disassembly of focal adhesions [39]. FAK is an important molecule that acts as a relay station for signals mediated by integrins or receptor tyrosine kinase, such as cytoskeletal changes, lamellipodia formation, cell proliferation and motility [40]. Aberrant activation of FAK-mediated signaling enhances cell proliferation, invasion, and metastasis [41]. Previous studies showed that aberrant expression of *PXN* was detected in a wide range of cancers, and its overexpression was usually correlated with worse prognosis of the patients [42]. However, in some types of cancers, downregulation of PXN was reported, e.g., breast cancer, colorectal cancer, leukemia, and low-grade glioma [43]. Here, we clearly showed that overexpression of *PXN* closely contributes to malignant transformation of HNSCC cells and a worse patient prognosis. It is an interesting finding that the expression of *PXN* differs depending on the type of cancer. Analysis of the epigenetic modification that regulates PXN expression is required for each cancer type. In HNSCC cells, analysis of *PXN* and *PXN*-mediated downstream signals will aid the search for therapeutic target molecules for this disease. 

Recent reports have shown that several dysregulated non-coding RNAs (long non-coding RNAs [lncRNAs] and miRNAs) are involved in aberrant expression of *PXN* in cancer cells [44,45,46]. Overexpressed lncRNAs were found to adsorb tumor-suppressor miRNAs and suppress their antitumor effects in cancer cells [47,48,49,50]. In gastric cancer, the lncRNA *XIST*, when overexpressed, adsorbed *miR-132* and caused upregulation of *PXN* in cancer cells. This study found that *XIST* acts as an oncogenic lncRNA in gastric cancer [51]. In triple-negative breast cancer, the lncRNA DLX6 antisense RNA 1 was upregulated in cancer tissues, and its expression promoted cell proliferation, EMT, and cisplatin resistance by regulating the *miR-199b-5p*/*PXN* axis [52]. These data support our current results. It is expected that many lncRNAs are involved in suppression of *miR-199* family expression in HNSCC cells.

A previous study showed that expression of *miR-218* was reduced by human papillomavirus-16 (HPV-16) in cervical cancer [53]. In HPV-16-infected OSCC cells, downregulation of *miR-218* and upregulation of *PXN* were detected, and these events contributed to colony formation and invasion of OSCC cells [54]. Moreover, low expression of *miR-218,* or high expression of *PXN,* was closely associated with overall survival and relapse-free survival [55]. Our previous studies demonstrated that *miR-218* acts as a tumor-suppressive miRNA in HNSCC by markedly suppressing cancer cell migration and invasive abilities [56]. Suppression of *miR-218* expression might be linked to aberrant expression of *PXN* in HNSCC cells.

Many non-coding RNA molecules are involved in the regulation of PXN expression, and the complexity of the molecular mechanisms of cancer cells has been clarified. To clarify the malignant transformation of HNSCC, genome-wide analysis including non-coding RNA molecules will be indispensable.

## 5. Conclusions

*PXN* was discovered by searching for oncogenic target genes of *miR-199*-5p and *miR-199-3p*, tumor suppressive miRNAs in HNSCC. Aberrant expression of *PXN* facilitated cancer cell migration and invasion, and its expression was closely associated with HNSCC molecular pathogenesis. Searching for target genes of tumor-suppressive miRNAs is an attractive strategy for exploring the molecular mechanism of HNSCC.

## Figures and Tables

**Table 1:** *miR-199-5p/3p* common target genes.

Entrez Gene ID	Gene Symbol	Gene Name	*miR-199-5p* Total Conserved Sites	*miR-199-3p* Total Conserved Sites
19	*ABCA1*	ATP-binding cassette, sub-family A (ABC1), member 1	1	1
340485	*ACER2*	alkaline ceramidase 2	1	1
92	*ACVR2A*	activin A receptor, type IIA	1	2
93	*ACVR2B*	activin A receptor, type IIB	3	1
57188	*ADAMTSL3*	ADAMTS-like 3	1	3
120	*ADD3*	adducin 3 (γ)	1	1
157	*ADRBK2*	adrenergic, beta, receptor kinase 2	1	1
81573	*ANKRD13C*	ankyrin repeat domain 13C	1	1
283373	*ANKRD52*	ankyrin repeat domain 52	1	1
57569	*ARHGAP20*	Rho GTPase activating protein 20	1	1
23365	*ARHGEF12*	Rho guanine nucleotide exchange factor (GEF) 12	1	1
222255	*ATXN7L1*	ataxin 7-like 1	1	1
27443	*CECR2*	cat eye syndrome chromosome region, candidate 2	2	1
10659	*CELF2*	CUGBP, Elav-like family member 2	1	2
387119	*CEP85L*	centrosomal protein 85kDa-like	1	1
153222	*CREBRF*	CREB3 regulatory factor	1	1
51232	*CRIM1*	cysteine rich transmembrane BMP regulator 1 (chordin-like)	1	1
1496	*CTNNA2*	catenin (cadherin-associated protein), α2	1	1
84301	*DDI2*	DNA-damage inducible 1 homolog 2 (*S. cerevisiae*)	1	1
55789	*DEPDC1B*	DEP domain containing 1B	1	1
2066	*ERBB4*	v-erb-b2 avian erythroblastic leukemia viral oncogene homolog 4	1	2
55137	*FIGN*	fidgetin	1	1
23767	*FLRT3*	fibronectin leucine rich transmembrane protein 3	1	1
10690	*FUT9*	fucosyltransferase 9 (α (1,3) fucosyltransferase)	1	1
8087	*FXR1*	fragile X mental retardation, autosomal homolog 1	2	1
2651	*GCNT2*	glucosaminyl (N-acetyl) transferase 2, I-branching enzyme (I blood group)	1	1
54891	*INO80D*	INO80 complex subunit D	1	1
3675	*ITGA3*	integrin, α3 (antigen CD49C, α 3 subunit of VLA-3 receptor)	1	1
8516	*ITGA8*	integrin, α8	1	1
11278	*KLF12*	Kruppel-like factor 12	1	1
26249	*KLHL3*	kelch-like family member 3	1	3
84458	*LCOR*	ligand dependent nuclear receptor corepressor	2	2
10960	*LMAN2*	lectin, mannose-binding 2	1	1
84061	*MAGT1*	magnesium transporter 1	1	1
4217	*MAP3K5*	mitogen-activated protein kinase 5	1	1
5599	*MAPK8*	mitogen-activated protein kinase 8	1	1
90411	*MCFD2*	multiple coagulation factor deficiency 2	1	1
54842	*MFSD6*	major facilitator superfamily domain containing 6	1	1
51701	*NLK*	nemo-like kinase	3	1
57532	*NUFIP2*	nuclear fragile X mental retardation protein interacting protein 2	1	1
10298	*PAK4*	p21 protein (Cdc42/Rac)-activated kinase 4	1	2
27253	*PCDH17*	protocadherin 17	1	1
5150	*PDE7A*	phosphodiesterase 7A	1	1
57475	*PLEKHH1*	pleckstrin homology domain containing, family H (with MyTH4 domain) member 1	1	1
5495	*PPM1B*	protein phosphatase, Mg^2+^/Mn^2+^ dependent, 1B	1	1
55607	*PPP1R9A*	protein phosphatase 1, regulatory subunit 9A	1	1
63976	*PRDM16*	PR domain containing 16	1	1
5813	*PURA*	purine-rich element binding protein A	1	1
5829	*PXN*	paxillin	1	1
5925	*RB1*	retinoblastoma 1	1	1
54502	*RBM47*	RNA binding motif protein 47	1	1
5991	*RFX3*	regulatory factor X, 3 (influences HLA class II expression)	1	1
6096	*RORB*	RAR-related orphan receptor B	1	2
9644	*SH3PXD2A*	SH3 and PX domains 2A	1	1
25769	*SLC24A2*	solute carrier family 24 (sodium/potassium/calcium exchanger), member 2	1	1
8303	*SNN*	stannin	1	1
6667	*SP1*	Sp1 transcription factor	1	1
257397	*TAB3*	TGF-β activated kinase 1/MAP3K7 binding protein 3	1	1
57551	*TAOK1*	TAO kinase 1	1	2
10099	*TSPAN3*	tetraspanin 3	1	1
57695	*USP37*	ubiquitin specific peptidase 37	1	1
23063	*WAPAL*	wings apart-like homolog (*Drosophila*)	1	2
10472	*ZBTB18*	zinc finger and BTB domain containing 18	1	2
26137	*ZBTB20*	zinc finger and BTB domain containing 20	2	2
6935	*ZEB1*	zinc finger E-box binding homeobox 1	1	1
80139	*ZNF703*	zinc finger protein 703	1	1
374655	*ZNF710*	zinc finger protein 710	1	1
283337	*ZNF740*	zinc finger protein 740	1	1

**Table 2:** Gene set enrichment analysis.

A. Significantly Enriched Gene Sets in the High *FXR1* Expression Group		
Name	Normalized Enrichment Score	FDR *q*-Value
KEGG_Cardiac muscle contraction	2.009	0.001
KEGG_Dilated cardiomyopathy	1.968	0.001
KEGG_Hypertrophic cardiomyopathy HCM	1.929	0.003
KEGG_Maturity onset diabetes of the young	1.918	0.002
**B. Significantly enriched gene sets in the high *PXN* expression group**		
**Name**	**normalized enrichment score**	**FDR *q*-value**
KEGG_Focal adhesion	2.458	*q* < 0.001
KEGG_ECM receptor interaction	2.316	*q* < 0.001
KEGG_Cytosolic DNA sensing pathway	2.117	0.001
KEGG_Proteosome	2.029	0.003
KEGG_Hypetrophic cardiomyopathy HCM	1.995	0.005
KEGG_NOD-like receptor signaling pathway	1.896	0.009
KEGG_Viral myocarditis	1.894	0.008
KEGG_Dilated Cardiomyopathy	1.804	0.014
KEGG_Bladder cancer	1.784	0.016
KEGG_Cytokine cytokine receptor interaction	1.776	0.016
KEGG_JAK/STAT signaling pathway	1.712	0.026
KEGG_RIG-I-like receptor signaling pathway	1.673	0.033
KEGG_Small cell lung cancer	1.644	0.038
KEGG_Arryhythmogenic right ventricular cardiomyopathy ARVC	1.639	0.037

## Data Availability

The results shown here are, in part, based upon data generated by the TCGA Research Network: https://www.cancer.gov/tcga (accessed on 10 April 2020). The data presented in this study are available on request from the corresponding author.

## Supplementary Materials

The following are available online at https://www.mdpi.com/article/10.3390/genes12121910/s1. Figure S1: Chromosomal locations and mature sequences of the *miR-199* family. Figure S2: The expression levels of *miR-199* family. Figure S3: Functional assays following ectopic expression of *miR-199-5p* and *miR-199-3p*, Figure S4: Photomicrographs of cells subjected to invasion assays. Figure S5: Photomicrographs of cells subjected to wound healing assays. Figure S6: Correlations between *miR-199* family and putative target genes. Figure S7: Full-size images of the Western blots shown in Figure 5. Figure S8: Efficiency of siRNA-mediated *PXN* knockdown in HNSCC cell lines (SAS and Sa3 cells). Figure S9: Photomicrographs of cells subjected to invasion assays. Figure S10: Photomicrographs of cells subjected to wound healing assays. Table S1: Features of HNSCC cell lines. Table S2: Reagents used in this study. Table S3: Clinical features of three HNSCC cases used for immunohistochemical staining.
