# Supplementary material for: Identification of miR-199-5p and miR-199-3p Target Genes: Paxillin Facilities Cancer Cell Aggressiveness in Head and Neck Squamous Cell Carcinoma
Source: Genes (Basel). 2021 Nov 27;12(12):1910. doi: 10.3390/genes12121910 (PMC8701835; doi:10.3390/genes12121910)
Supplement: Supplementary file 1 [file genes-12-01910-s001.zip › genes-1436889 - Supplementary Figures.pdf]

Figure S1

A

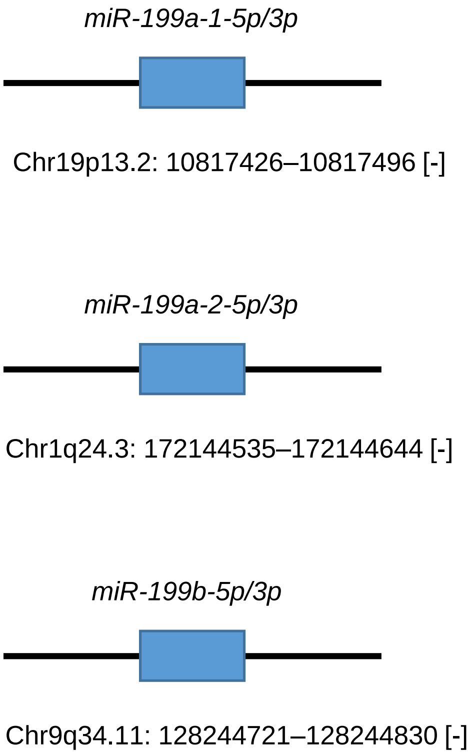

B

The guide strands

miR-199a-1-5p	CCCAGUGUUCAGACUACCUGUUC
miR-199a-2-5p	CCCAGUGUUCAGACUACCUGUUC
miR-199b-5p	CCCAGUGUUUAGACUAUCUGUUC

The passenger strands

miR-199a-1-3p	ACAGUAGUCUGCACAUUGGUUA
miR-199a-2-3p	ACAGUAGUCUGCACAUUGGUUA
miR-199b-3p	ACAGUAGUCUGCACAUUGGUUA

Supplementary Figure S1: Chromosomal locations and mature sequences of the *miR-199* family.
(A) The *miR-199* family is composed of 3 species: *miR-199a-1*, *miR-199a-2*, and *miR-199b* located on human chromosome 19p13.2, 1q24.3, and 9q34.11 respectively. (B) Mature sequences of the *miR-199* family are shown. miRBase shows that *miR-199-5p* annotates as the guide strand and *miR-199-3p* as the passenger strand. Mature sequences of *miR-199a-1-5p* and *miR-199a-2-5p* are identical. The mature sequence of miR-*199b-5p* differs in one base compares with *miR-199a-5p*. The seed sequences of the 3 miRNAs are identical (red letters). Mature sequences of *miR-199a-1-3p*, *miR-199a-2-3p* and *miR-199b-3p* are identical. The seed sequences of the 3 miRNAs are identical (blue letters).

Figure S2

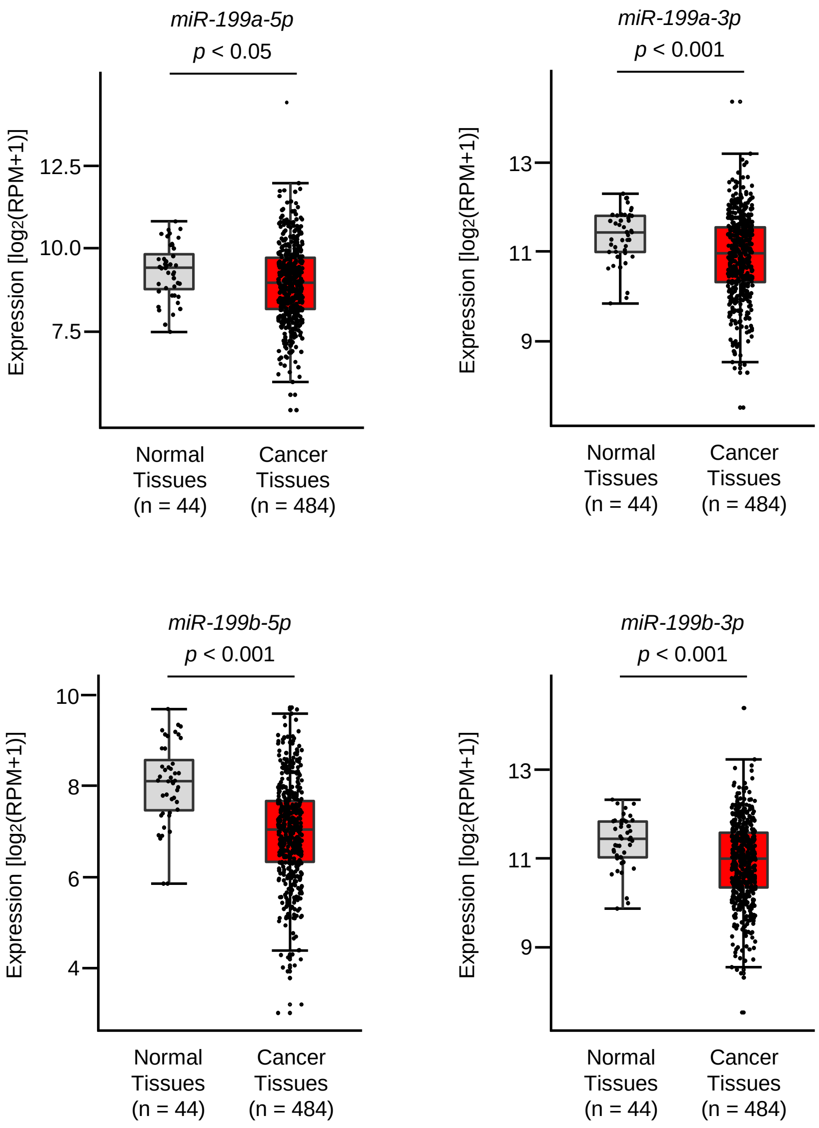

Supplementary Figure S2: The expression levels of *miR-199* family.
The *miR-199* family (*miR-199a-5p*, *miR-199a-3p*, *miR-199-b-5p*, and *miR-199b-3p*) were evaluated by TCGA-HNSC dataset. All member of *miR-199* family were downregulated in cancer tissues.

Figure S3

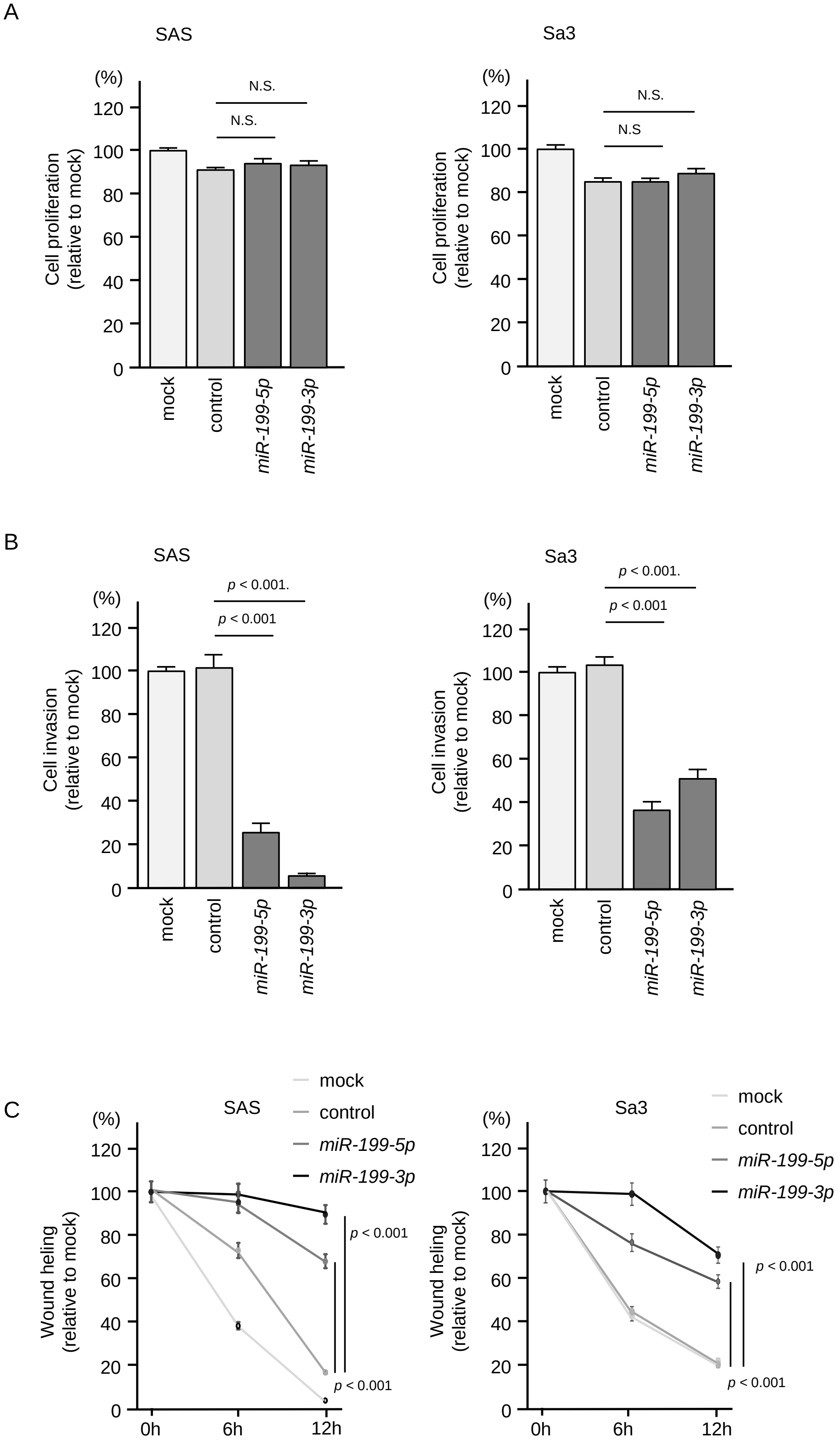

Supplementary Figure S3: Functional assays of cell proliferation, invasion and migration following ectopic expression of *miR-199-5p* and *miR-199-3p* in HNSCC cell lines (SAS and Sa3).
(A) Cell proliferation assessed by XTT assay at 72 h after *miR-199-5p* and *miR-199-3p* transfection (N.S., not significant). (B) Cell invasion assessed by Matrigel invasion assays at 48 h after seeding *miR-199-5p* and *miR-199-3p* transfected cells into chambers. (C) Cell migration assessed by wound healing assay at 0, 6, and 12 h after cell scratch formation.

Figure S4

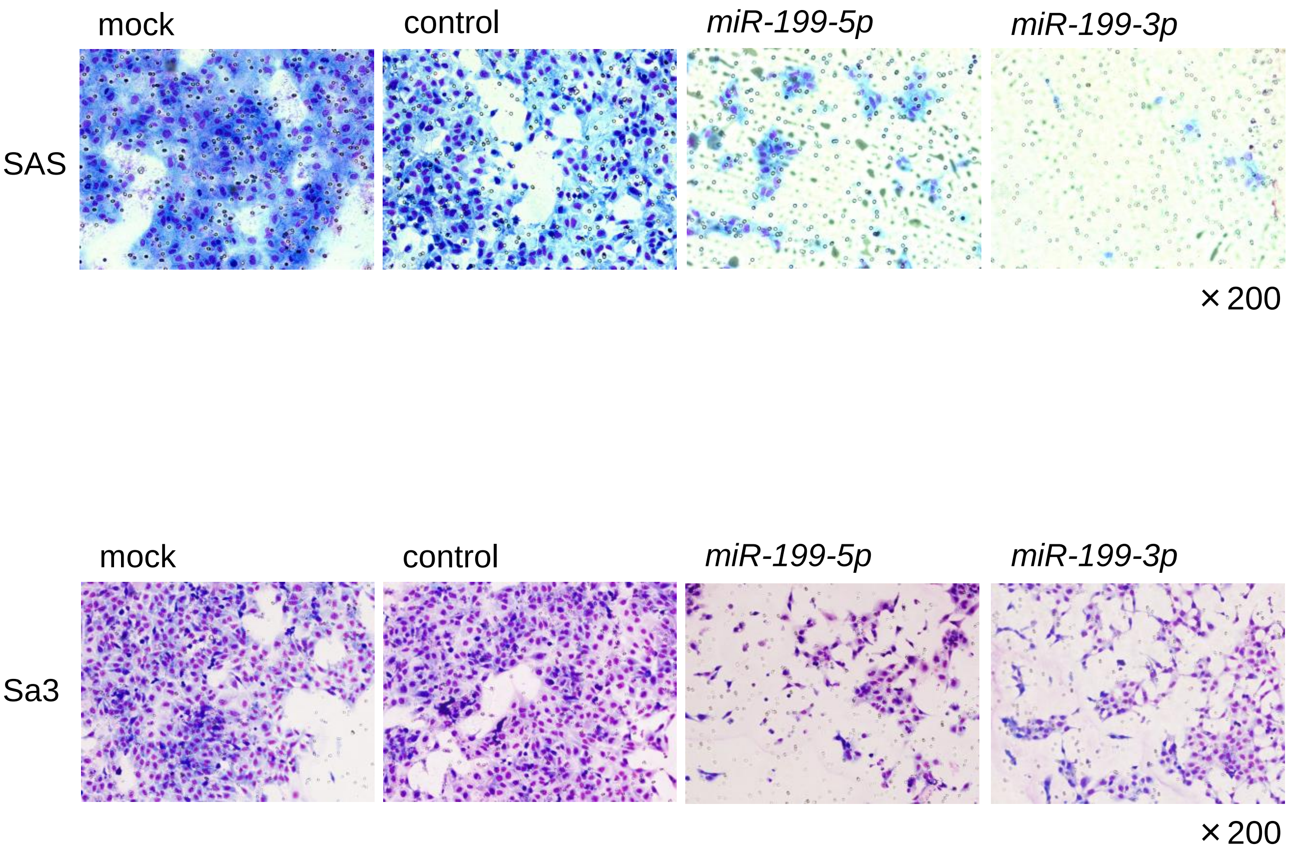

Supplementary Figure S4: Photomicrographs of cells subjected invasion assays.
Typical images of cells in invasion assays following *miR-199-5p* and *miR-199-3p* transfection into HNSCC cell lines, SAS and Sa3.

Figure S5

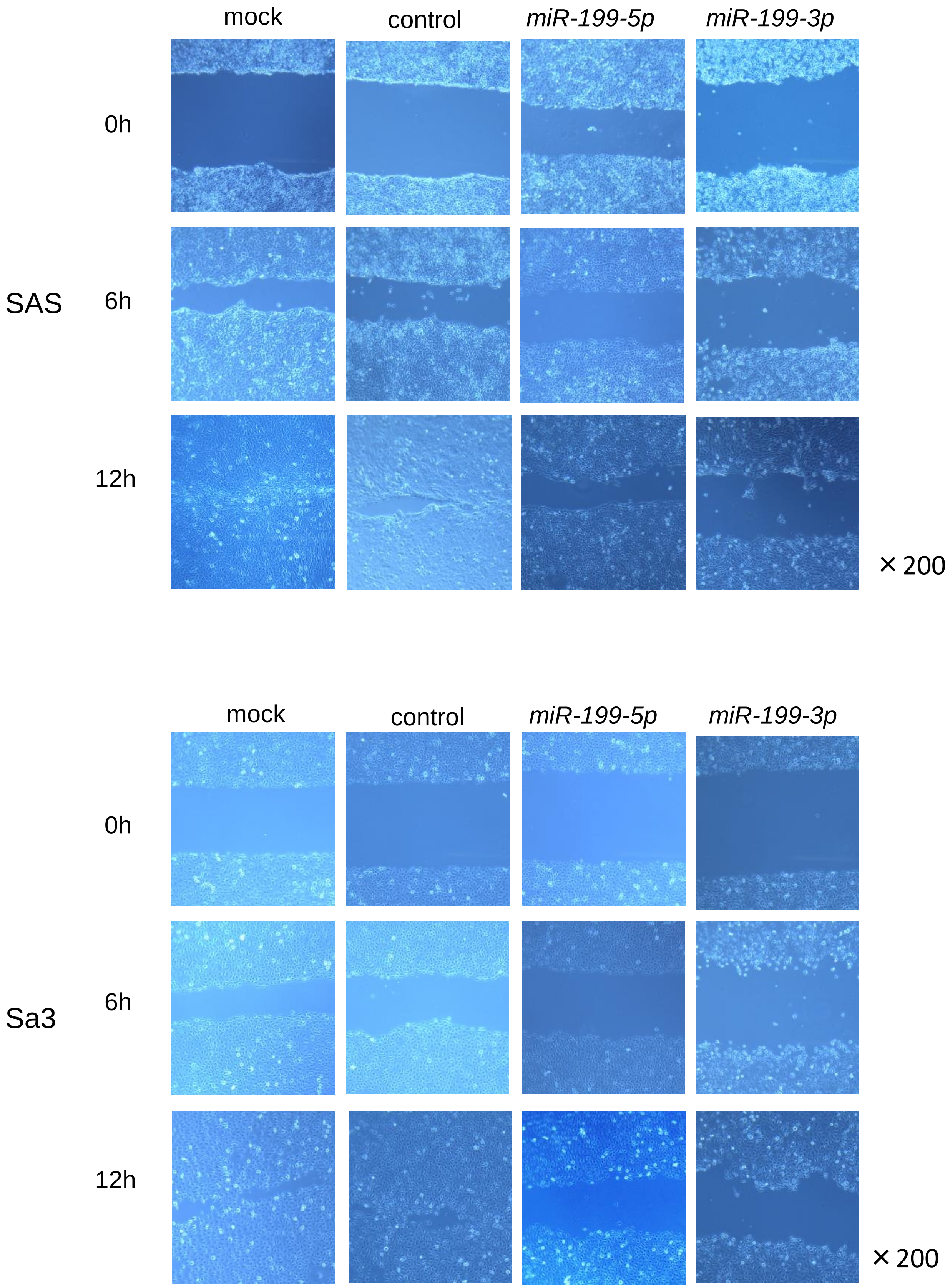

Supplementary Figure S5: Photomicrographs of cells subjected wound heling assays.
Typical images of cells in wound heling assays following *miR-199-5p* and *miR199-3p* transfection into HNSCC cell lines, SAS and Sa3.

Figure S6

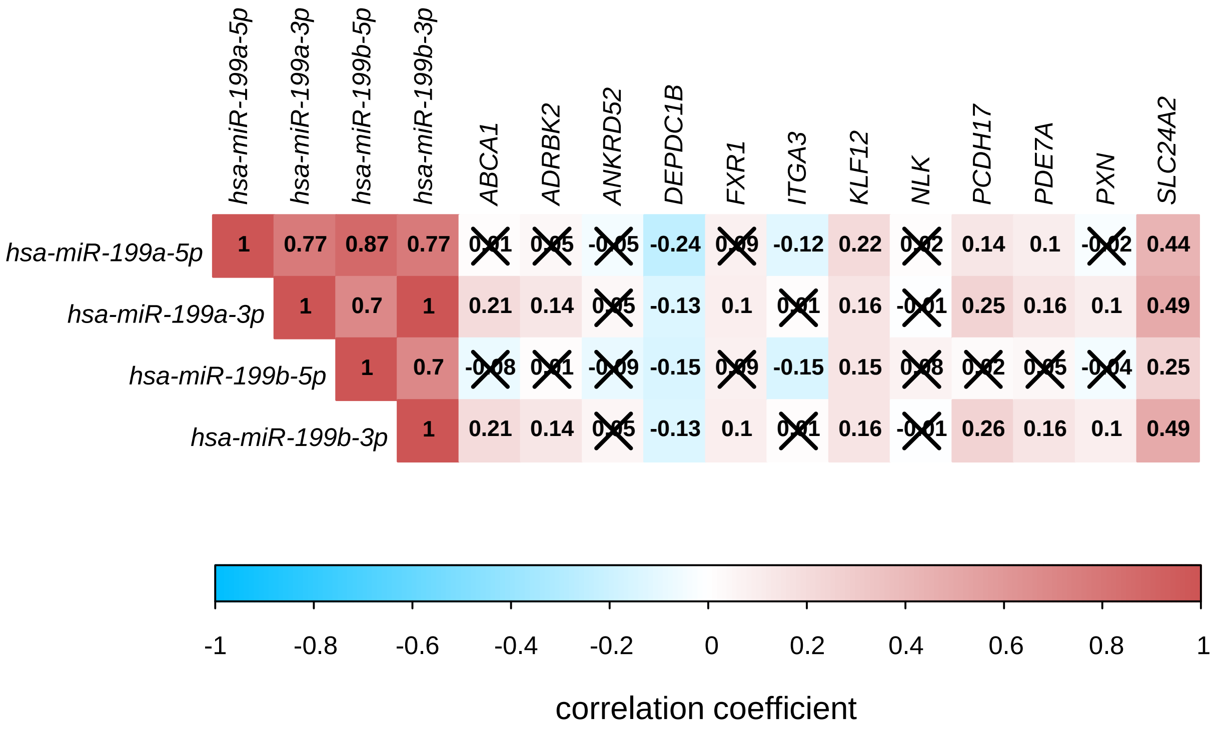

Supplementary Figure S6: Correlation between *miR-199* family and putative target genes in TCGA-HNSC. The plots showed the correlation among expression levels of *miR-199* family and putative target genes in cancer tissues from TCGA-HNSC according to Spearman's rank tests. The values in each cell showed correlation coefficients between miRNA/gene in each row and column. The color scale was created by correlation values. Red cells indicated positive , and blue cells indicated negative correlation. Cells without a cross represented that a relationship was significant (*p*-value < 0.05).

Figure S7

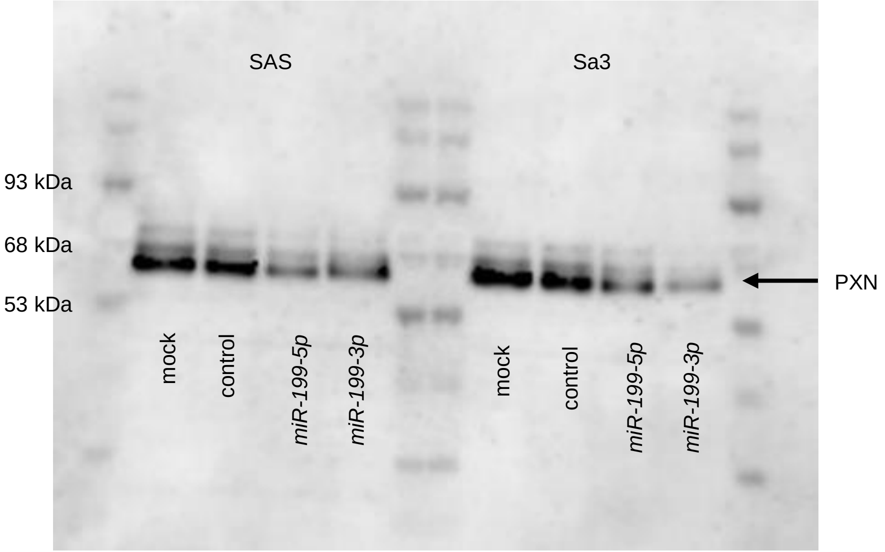

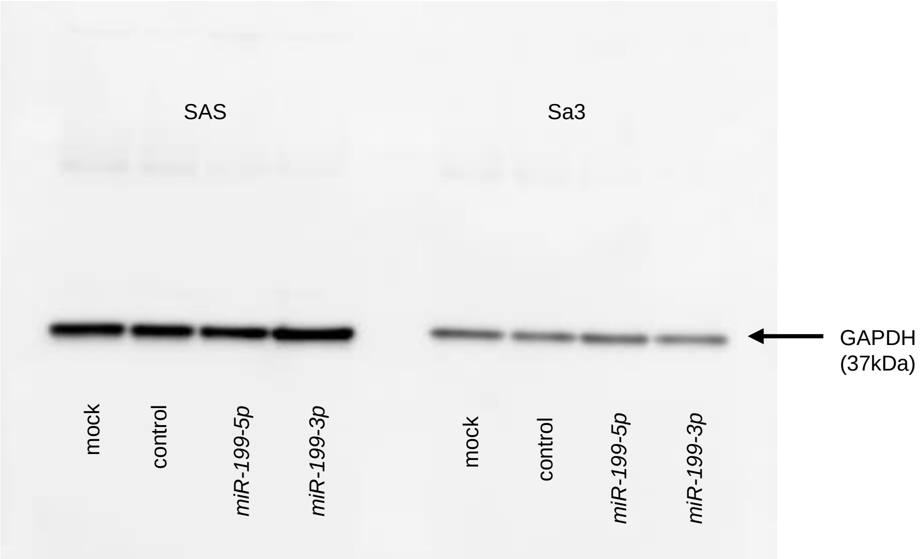

Supplementary Figure S7: Full size image of Western blotting of Figure 5.

Figure S8

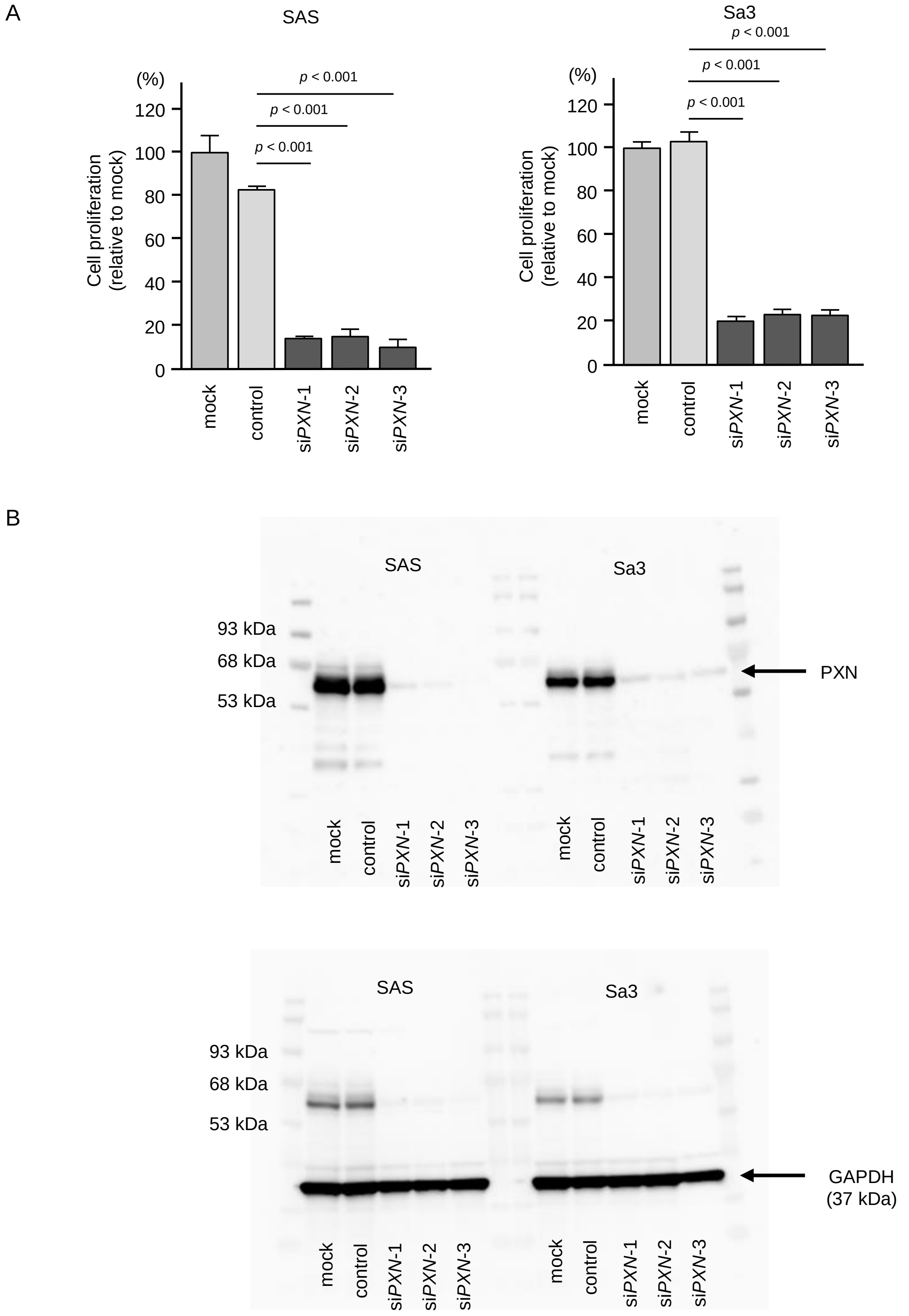

Supplementary Figure S8: siRNA knockdown efficiencies for *PXN* in HNSCC cell lines (SAS and Sa3 cells).

The efficiencies of PXN knockdown by si*PXN*-1, si*PXN*-2 and si*PXN-3* were evaluated by qRT-PCR (A) and Western blotting (B). The *PXN* mRNA and protein levels were measured 72 h after siRNA transfection. GAPDH mRNA and protein levels were used as internal controls for qRT-PCR and Western blotting, respectively.

Figure S9

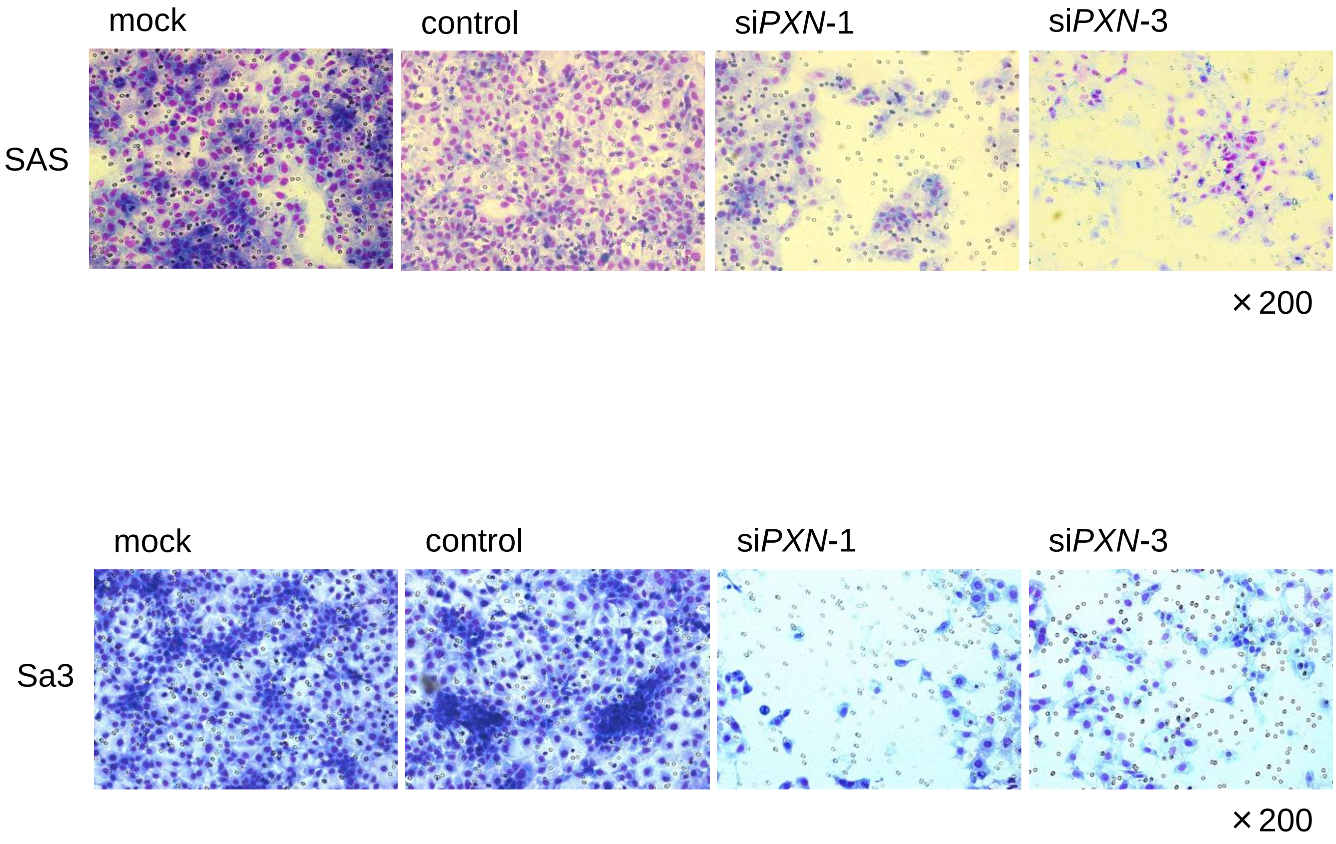

Supplementary Figure S9: Photomicrographs of cells subjected invasion assays.
Typical images of cells in invasion assays following si*PXN*-1 and si*PXN*-3 transfection into HNSCC cell lines, SAS and Sa3.

Figure S10

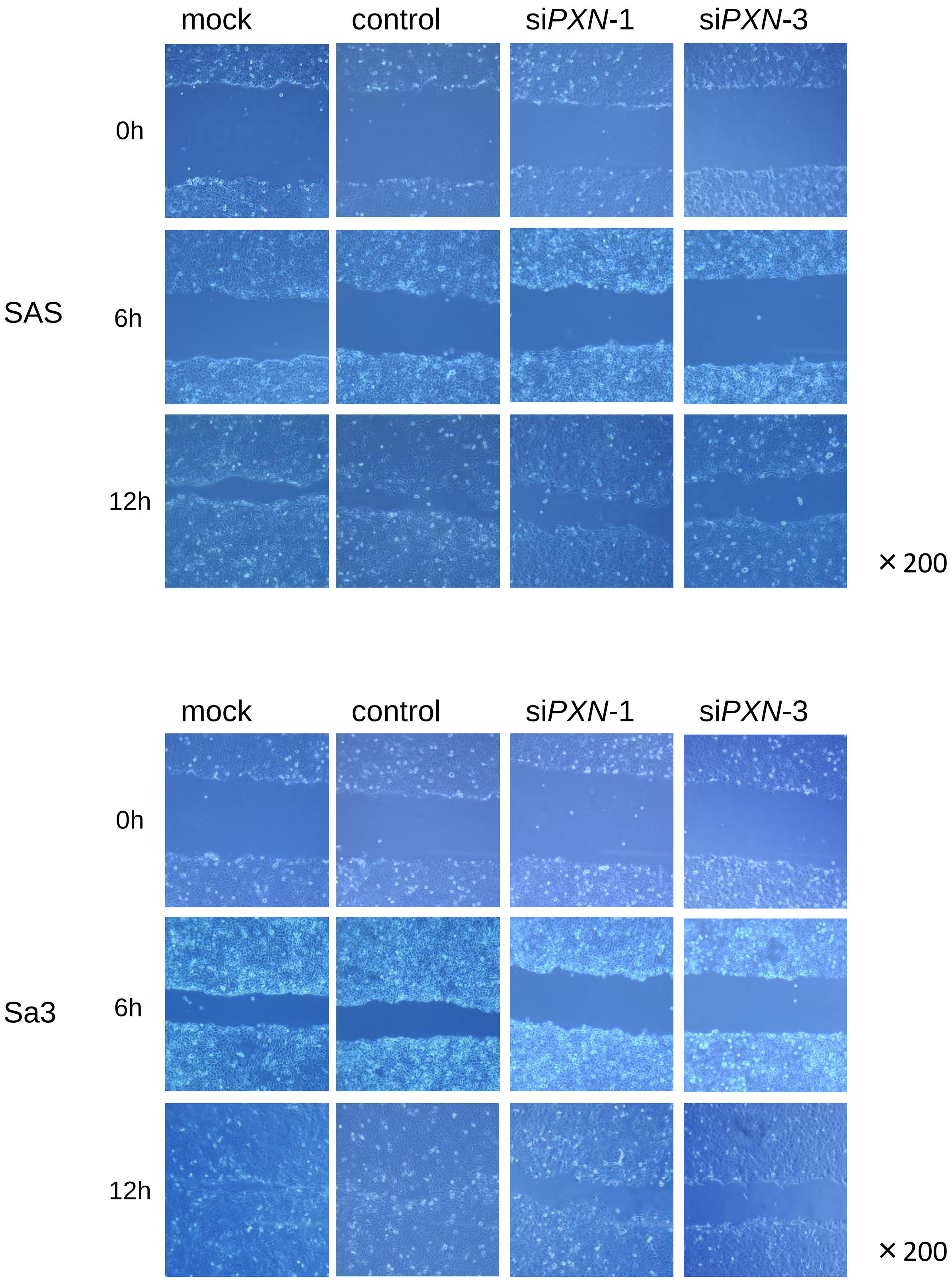

Supplementary Figure S10: Photomicrographs of cells subjected wound heling assays.
Typical images of cells in wound heling assays following si*PXN*-1 and si*PXN*-3 transfection into HNSCC cell lines, SAS and Sa3.
